# Anharmonicity in Molecular Crystals: Generalized Perturbation Theory Meets Periodic Computations

**DOI:** 10.1021/acs.jpclett.5c02217

**Published:** 2025-09-15

**Authors:** Davide Mitoli, Alessandro Erba, Vincenzo Barone, Marco Mendolicchio

**Affiliations:** † 9314 Università di Torino, Via Pietro Giuria 7, 10125 Torino, Italy; ‡ 563196 INSTM, via G. Giusti 9, 50121 Firenze, Italy; ¶ 19004 Scuola Normale Superiore, Piazza dei Cavalieri 7, 56126 Pisa, Italy

## Abstract

Accurate simulation of vibrational spectra in the solid state remains a major challenge due to the combined effects of anharmonicity, intermolecular interactions, and resonance phenomena. In this work, we introduce a generalized second-order vibrational perturbation theory (GVPT2) framework for the quantitative computational spectroscopy of molecular solids. The method balances efficiency and accuracy through a perturb-then-diagonalize approach in which resonant terms are excluded in the initial perturbative treatment and subsequently handled more accurately through a variational approach. This strategy ensures numerical stability while capturing essential vibrational couplings. As a representative application, we investigated the infrared spectrum of solid carbon dioxide (dry ice), a prototypical system exhibiting strong anharmonic effects and Fermi resonances. The generalized VPT2 approach accurately reproduces both absolute band positions and splitting patterns, yielding results in excellent agreement with the experimental data. These findings demonstrate the potential of the method for reliable and transferable anharmonic vibrational analysis across a broad class of solid-state systems.

## Graphical abstract

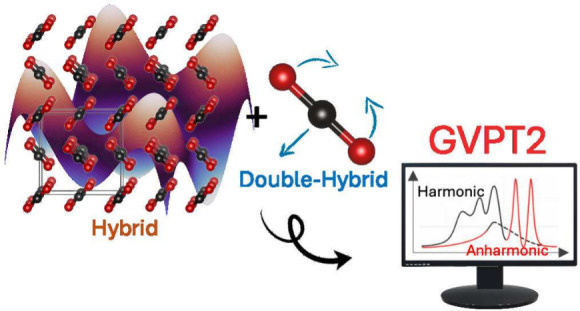

Vibrational spectroscopy is a key tool for exploring molecular structure, dynamics, and interactions, providing direct access to critical regions of the potential energy surface (PES) that underlie the structural and dynamic properties of molecular systems. However, interpreting the experimental outcome greatly benefits from the support of accurate quantum-chemical (QC) calculations, which allow one to assign spectral features, identify couplings, and extract structural parameters. While numerous theoretical approaches going beyond the harmonic approximation have been successfully developed and validated for isolated gas-phase molecules, the situation is considerably more complex in the solid state. In this context, anharmonic effects, intermolecular interactions, and symmetry breaking pose significant challenges for both experimental and theoretical investigations.

From a computational point of view, the Crystal package has recently been extended with methodologies accounting for anharmonic effects.^1–4^ Two main factors may still hinder accurate predictions of absolute band positions and intensities for complex materials: (i) the vibrational problem is tackled using the vibrational configuration interaction (VCI) approach,^5–7^ based either on harmonic basis functions or on a preliminary vibrational self-consistent field (VSCF) treatment,^8^ which becomes computationally prohibitive as the number of vibrational modes increases; (ii) the PES is described within the density functional theory (DFT), with density-functional approximations (DFAs) of the first four rungs of “Jacob’s ladder” (LDA, GGA, meta-GGA, and hybrid),^9^ while correlated wave function-based electronic structure methods and/or DFAs from the fifth rung of the ladder, known to provide a more accurate description of the PES for isolated molecules,^10,11^ are not readily available.

To address this gap, we propose a robust and computationally affordable theoretical framework for the simulation of vibrational spectra of molecular solids using solid carbon dioxide (dry ice) as a paradigmatic case study. This system is particularly suitable due to its pronounced anharmonicity and the presence of strong Fermi resonance (FR) effects. A recent study presented a detailed analysis of the main FR in the Raman spectrum of dry ice, offering an unbiased assignment of the experimental signatures along with a theoretical description of two-mode and three-mode coupling mechanisms underlying the observed dyad.^12^ Although the computed splittings involving the relevant states showed remarkable agreement with the experimental data, quantitative agreement for absolute band positions was not achieved.

To tackle this limitation, we resort to second-order vibrational perturbation theory (VPT2),^13–18^ which offers an appealing balance between accuracy and computational cost, making it particularly suitable for medium- to large-sized chemical systems. Although VPT2 is often regarded as inadequate in the presence of strong resonances, its generalized formulation, adopted in this work, overcomes such limitations by implementing a “perturb-then-diagonalize” approach.^19,20^ In this scheme, resonant couplings are excluded from the initial perturbative treatment and reintroduced through a subsequent variational step, thus recovering essential interactions without compromising the numerical stability. Notably, VPT2 yields exact results for vibrational levels of modes well described by Morse-like potentials.

While we refer interested readers to refs 13, 17, 18, and 20–23 for more details, an overview of the VPT2 framework is provided below, with emphasis on the treatment of resonances. To this end, let us consider a system characterized by *N* active modes. The VPT2 Hamiltonian can be expressed as follows

1 $$H=\frac{1}{2}\overset{N}{\underset{i=1}{\sum }}\omega_{i}\left({q_{i}}^{2}+{p_{i}}^{2}\right)+\frac{1}{6}\overset{N}{\underset{i=1}{\sum }}\overset{N}{\underset{j=1}{\sum }}\overset{N}{\underset{k=1}{\sum }}\eta_{ijk}q_{i}q_{j}q_{k}+\frac{1}{24}\overset{N}{\underset{i=1}{\sum }}\overset{N}{\underset{j=1}{\sum }}\overset{N}{\underset{k=1}{\sum }}\overset{N}{\underset{l=1}{\sum }}\eta_{ijkl}q_{i}q_{j}q_{k}q_{l}$$

where ω_*i*_ and *q*_*i*_ are the *i*th harmonic wavenumber (in cm^–1^) and dimensionless normal coordinate, while η_*ijk*_ and η_*ijkl*_ represent, respectively, the third- and fourth-order derivatives of the PES ( V ), introduced through the following closed-form notation:

2 $$\eta_{ijk...}={\left(\frac{\partial^{n}V}{\partial q_{i}\partial q_{j}\partial q_{k}...}\right)}_{\mathrm{eq}}$$

The anharmonic vibrational energies can be derived through both the Rayleigh–Schrödinger (RSPT)^24^ and Canonical Van Vleck (CVPT)^25^ perturbation theories, yielding the same expression valid for any vibrational level. In particular, the transition energy ν_*R*_ from the ground state to a generic vibrational state *R* can be expressed as

3 $$\nu_{R}=\epsilon_{R}-\epsilon_{0}=\overset{N}{\underset{i=1}{\sum }}\omega_{i}v_{R,i}+\overset{N}{\underset{i=1}{\sum }}\overset{N}{\underset{j=i}{\sum }}\chi_{ij}\left[v_{R,i}v_{R,j}+\frac{1}{2}\left(v_{R,i}+v_{R,j}\right)\right]$$

where *v*_*R*_ = *v*_*R*,1_, ..., *v*_*R*,*N*_ is the vector that collects the vibrational quantum numbers of the state *R*, ϵ_0_ is the resonance-free zero-point vibrational energy (ZPVE), and the elements of the matrix **χ** are given below:

4 $$16\chi_{ii}=\eta_{iiii}-\frac{5{\eta_{iii}}^{2}}{3\omega_{i}}-\overset{N}{\underset{\begin{array}{c} j=1\\ \left(j\neq i\right) \end{array}}{\sum }}\frac{{\eta_{iij}}^{2}\left(8{\omega_{i}}^{2}-3{\omega_{j}}^{2}\right)}{\omega_{j}\left(4{\omega_{i}}^{2}-{\omega_{j}}^{2}\right)}$$

$$\begin{array}{l} 4\chi_{ij}=\eta_{iijj}-\frac{2{\eta_{iij}}^{2}\omega_{i}}{4{\omega_{i}}^{2}-{\omega_{j}}^{2}}-\frac{2{\eta_{ijj}}^{2}\omega_{j}}{4{\omega_{j}}^{2}-{\omega_{i}}^{2}}-\frac{\eta_{iii}\eta_{ijj}}{\omega_{i}}-\frac{\eta_{iij}\eta_{jjj}}{\omega_{j}}-\\ \overset{N}{\underset{k=1\left(k\neq i,j\right)}{\sum }}\left[\frac{2\omega_{k}\left({\omega_{k}}^{2}-{\omega_{i}}^{2}-{\omega_{j}}^{2}\right){\eta_{ijk}}^{2}}{\left(\omega_{i}+\omega_{j}+\omega_{k}\right)\left(\omega_{i}-\omega_{j}-\omega_{k}\right)\left(\omega_{i}-\omega_{j}+\omega_{k}\right)\left(\omega_{i}+\omega_{j}-\omega_{k}\right)}+\frac{\eta_{iik}\eta_{jjk}}{\omega_{k}}\right] \end{array}$$ 5

As is well recognized, eqs 4 and 5 suffer from the presence of potentially vanishing denominators when 2ω_*i*_ ≈ ω_*j*_ and ω_*i*_ ≈ ω_*j*_ + ω_*k*_, commonly referred to as FRs of types I and II, respectively. Over the last few decades, different approaches have been developed to tackle this problem, most of them relying on a two-step procedure: the terms of interest are systematically screened, and for each pair of states, the energetic proximity is estimated. If the latter is below a specified threshold (200 cm^–1^ in the present work), then the “weight” of the term is estimated. In this work, the test proposed by Martin and co-workers^26^ has been adopted. Within this approach, the difference between the perturbative energies and those arising from *ad hoc* variational models is estimated, and if this difference exceeds a second threshold (typically set to 1 cm^–1^), then the term is marked as resonant. The terms labeled as resonant can then be systematically removed, with the resulting method being known as deperturbed VPT2 (DVPT2).

An alternative strategy is the so-called degeneracy-corrected PT2 (DCPT2),^27^ in which each potentially resonant term is replaced *a priori* by a nonresonant contribution derived from a Taylor series expansion

6 $$\frac{Sk^{2}}{2\epsilon }\to S\left(\sqrt{k^{2}+\epsilon^{2}}-\epsilon \right)$$

where ϵ is half the absolute frequency difference, *k* ^2^ is the constant term, and *S* is the sign (±1). Although this method avoids the need for numerical criteria in identifying FRs, it may suffer from a loss of accuracy when the system is far from resonance. To overcome this limitation, the hybrid degeneracy-corrected PT2 (HDCPT2)^28^ method was introduced. This approach allows for a smooth transition between VPT2 and DCPT2, depending on the nature of each term in eqs 5 and 6, and is currently the reference method for thermochemical applications.

While the DVPT2 method enables the removal of terms leading to unphysical transition energies, the corresponding truncation may also eliminate relevant interactions between vibrational states, potentially compromising accuracy. The so-called generalized VPT2 (GVPT2) introduces a further variational step to recover such non-negligible interactions. In this framework, a variational matrix **H**_var._ is constructed, with DVPT2 energies on the diagonal and the interaction terms of the contact-transformed Hamiltonian coupling resonant states included as off-diagonal elements.

At this stage, not only FRs but also Darling–Dennison resonances (DDRs)^29^ are considered. The latter are typically classified according to the number of quanta involved, including resonances of types 1–1, 2–2, and 1–3. DDRs do not directly affect the matrix **χ** but can induce significant interactions (e.g., involving the fundamentals of X–H stretching modes). The variational matrix is then diagonalized

7 $$H_{\mathrm{var}}L_{\mathrm{var}}=L_{\mathrm{var}}\epsilon_{\mathrm{var}}$$

where the diagonal elements of **ϵ**_var._ represent the new anharmonic transition energies, while the matrix **L**_var._ collects the eigenvectors and hence defines the variational states in terms of the harmonic ones.

The final step required to simulate the vibrational spectrum is the calculation of the intensities. In particular, the infrared intensity of a transition from the ground state to the generic state *R* is related to the corresponding transition moment **μ**_*R*,0_:

8 $$I_{R}\propto |\mu_{R,0}{|}^{2}\mathrm{with}\mu_{R,0}=\frac{\left\langle \psi_{R}\right|\mu \left|\psi_{0}\right\rangle }{\sqrt{\left\langle \psi_{R}\right|\psi_{R}\rangle }}$$

In this work, the wave functions are expanded up to second order and combined with a linear expansion of the dipole moment operator, as implemented in the Crystal software through a coupled-perturbed approach^30^

9 $$\mu \approx \mu^{\mathrm{eq}}+\overset{N}{\underset{i=1}{\sum }}\mu_{i}q_{i}$$

where **μ** ^eq^ and **μ**_*i*_ represent the equilibrium value of the dipole moment and its first-order derivative with respect to *q*_*i*_, respectively. The expressions describing anharmonic transition moments within the VPT2 framework (see the Supporting Information and refs 21 and 31–33 for further details), can be affected by both FR and DDR, so the previously described methodologies can also be used to compute their deperturbed counterparts. When the GVPT2 scheme is adopted, the deperturbed transition moments are then rotated according to the eigenvectors of the variational matrix

10 $$\mu^{\mathrm{GVPT}2}=L_{\mathrm{var}}^{†}\mu^{\mathrm{DVPT}2}$$

where **μ** ^GVPT2^ and **μ** ^DVPT2^ represent, respectively, the vectors collecting the GVPT2 and DVPT2 transition moments for all vibrational states included in the simulation. The resulting transition moments serve as the starting points for the calculation of vibrational intensities, including the variational correction.

The entire framework has been implemented as an extension of a program under development in our research group which is designed for (ro-)­vibrational analyses of chemical systems, as discussed elsewhere.^34^ The advantage of employing a local expansion of the potential energy in the solid state lies in the fact that once both the PES and the property surface (PS) have been parametrized, the anharmonic vibrational analysis can be approached in exactly the same way as for isolated molecules. The new program extracts harmonic frequencies, anharmonic force constants, and first-order derivatives of the dipole moment from a standard Crystal output file^35^ and applies a perturbative approach to compute transition energies and intensities via the previously introduced VPT2 schemes. The external program has been extended to enable anharmonic calculations in solid-state chemistry, employing either curvilinear or Cartesian coordinates through generalized VPT2 equations recently introduced by some of the present authors. Building on the data extracted from the Crystal output, VPT2 is applied to obtain anharmonic transition energies and intensities, while the dual-level method (vide infra) is realized by combining these with harmonic frequencies extracted from a standard Gaussian 16 output. The developed computational engine has been systematically employed for all the calculations reported in this work and is sketched in Figure S1 of the Supporting Information (SI).

The experimental IR spectrum of dry ice has been the subject of several studies in recent years^36,37^ and was more recently investigated by Isokoski and co-workers,^38^ who identified three distinct spectral regions of interest. The first exhibits a doublet structure with a splitting of approximately 5 cm^–1^, associated with the fundamental excitation of the bending mode. A sharp peak appears at around 2344 cm^–1^, corresponding to the fundamental excitation of the asymmetric stretching mode, while the symmetric stretching mode remains IR-inactive. A weaker peak attributable to the ^13^C isotopologue is also observed. Finally, two peaks above 3500 cm^–1^ have been assigned to combination bands involving the fundamentals of the asymmetric stretching mode together with the first overtone of the bending mode and the fundamental of the symmetric stretching mode.

Harmonic and anharmonic calculations on the solid have been carried out using a development version of the Crystal package.^35,39^ Dry ice has been modeled in the cubic phase with space group *Pa* 3, comprising four molecules per unit cell. The reciprocal space has been sampled using a 6 × 6 × 6 Monkhorst–Pack mesh, yielding 24 **k** points in the symmetry-irreducible portion of the first Brillouin zone. The calculations have been performed by combining the PBE functional with the pob-TZVP-rev2 triple-ζ basis set, specifically developed for solid-state quantum-chemical computations,^40^ and by systematically including empirical dispersion corrections (D3).^41,42^ From now on, this level of theory will be simply referred to as PBE. Harmonic and anharmonic force constants have been obtained via finite differences of analytical energies and gradients (EGH approach) within the 3M4T representation of the PES.^1,4^

The resulting quantities have then been processed by a standalone module to perform the perturbative treatment. Finally, the fundamental anharmonic frequencies of dry ice have been computed using different VPT2 schemes and are reported in Table 1. As suggested by the resulting values, only the symmetric stretching is significantly affected by the presence of FRs due to its interaction with the first overtone of the bending mode (or two distinct singly excited bending modes). On the other hand, the bending and asymmetric stretching fundamentals are only slightly influenced by the presence of DDRs. The computed splitting associated with the bending mode dyad (∼5 cm^–1^) is in good agreement with its experimental counterpart, and the same holds for the symmetric stretching, whose corresponding splitting (∼120 cm^–1^) is carefully analyzed in ref 12. Despite this, the accuracy of the absolute anharmonic frequencies is still not satisfactory, with discrepancies of about 90 cm^–1^ compared with the experimental data, making the development of more refined techniques essential to achieving quantitative agreement. In general terms, any vibrational quantity of interest Ω can be decomposed into a purely harmonic contribution Ω^harm^ and its anharmonic correction *ΔΩ* ^anh^:

11 $$\Omega =\Omega^{\mathrm{harm}}+\Delta \Omega^{\mathrm{anh}}$$

The harmonic term is usually larger in magnitude, making its accuracy much more critical for quantitative purposes. Furthermore, at a given level of theory, the anharmonic correction is significantly more expensive than the harmonic term. These premises pave the way for so-called dual-level methods in which a higher level (HL) of theory is adopted for computing the harmonic contributions and a lower level (LL) is employed for the calculation of the anharmonic corrections. Since analytical first- and second-order derivatives for double-hybrid functionals and post-Hartree–Fock methods are not available within the Crystal software, a strategy based on the interplay between different QC packages has been devised. Due to the similarity between the vibrations of interest when switching from the solid state to the gas phase, the anharmonic frequencies of dry ice have been computed using the following expression

12 $$\nu_{R}=\omega_{R}^{S}+\Delta \omega_{R}^{G}+\Delta \nu_{R}^{S}$$

where ω_*R*_^S^ and *Δν*_*R*_^S^ are the harmonic frequencies and anharmonic corrections computed in the solid state at the PBE level of theory. The term *Δω*_*R*_^G^ denotes a harmonic correction computed for the isolated molecule of carbon dioxide through the Gaussian 16 package^43^

13 $$\Delta \omega_{R}^{G}=\omega_{R}^{G}\left(\mathrm{rDSD}\right)-\omega_{R}^{G}\left(\mathrm{PBE}\right)$$

where rDSD denotes the revDSD-PBEP86-D4 double-hybrid functional,^10^ used in conjunction with the aug-cc-pVTZ basis set,^44^ which is obtained from its standard cc-pVTZ-F12 (3F12) counterpart^45^ by removing the *d* functions on first-row atoms and replacing the two *f* functions on second- and third-row atoms with a single *f* function taken from the cc-pVTZ basis set.^44^ Previous analyses demonstrated the reliability of the revDSD-PBEP86-D4 functional in providing accurate estimates of vibrational transition energies and intensities.^46,47^

**Table 1:** Computed Harmonic and Anharmonic Fundamentals of Dry Ice (in cm^–1^) at the PBE Level of Theory

State^a^	Assignment^b^	Harm.	VPT2	DVPT2	DCPT2	HDCPT2	GVPT2^c^
|1_18–19_⟩	Bending	632	627	627	627	627	626 (100%)
|1_20–22_⟩		633	627	627	627	627	626 (100%)
|1_23–25_⟩		637	631	631	631	631	630 (100%)
|1_26–28_⟩	Sym. str.	1331	1363	1311	1358	1358	1341 (72%)
							1215 (28%)
|1_29_⟩		1332	1358	1308	1350	1350	1337 (70%)
							1216 (30%)
|1_30_⟩	Asym. str.	2349	2302	2302	2302	2302	2284 (99%)
|1_31–33_⟩		2364	2312	2312	2312	2312	2294 (99%)

a Vibrational fundamental states in ascending order of harmonic frequency. Only active modes are included.

b Assignment of the vibrations, classified as bending motions (bending), symmetric stretching (sym. str.), and asymmetric stretching (asym. str.).

c Anharmonic fundamental wavenumbers at the GVPT2 level. Percentage contributions of the principal states to each wave function are reported in parentheses.

This procedure has also been systematically applied to calculate the intensities associated with the fundamental bands. While the number of normal modes in solid-state calculations depends on the number of atoms per unit cell, that of the isolated molecule remains constant. As a result, the harmonic correction reported in eq 13 remains constant within a specific class of vibrations (bending, asymmetric stretching, and symmetric stretching). The expression given in eq 13 has been consistently used to refine the band positions, and the results corresponding to the observed bands at 15 K are reported in Table 2. The introduction of this harmonic correction leads to a significant improvement in the band positions, with the largest deviation (approximately 4 cm^–1^) affecting the fundamentals of the asymmetric stretching mode. At the same time, the transition frequencies closely match the experimental values even above 3500 cm^–1^, which is the region most influenced by the treatment of resonances. Notably, only within the GVPT2 framework is it possible to correctly reproduce the dyad observed in the experimental spectral profile. Specifically, the involved states |1_1_1_3_⟩ and |2_2_1_3_⟩ can equivalently be expressed as |*v* + 1_1_⟩ and |*v* + 2_2_⟩, with *v* = 1_1_. These two states participate in the well-known Fermi resonance that also affects the Raman spectrum. Consequently, its effects can be accurately captured only when explicitly treated at the variational level, in terms of both transition energies and intensities.

**Table 2:** Comparison between the Computed and Experimental (at 15 K) Observed Fundamental Spectroscopic Data for Dry Ice^a^

	Harm.			Anharm.		
	Solid	Gas		Solid	Hybrid	
Assignment	PBE	PBE	rDSD	PBE	rDSD//PBE	Exp.
CO_2_ bend (|1_2_⟩)	637(324)	644(28)	668(27)	630(288)	654(289)	659.72
	632(181)	644(28)	668(27)	625(208)	649(209)	654.53
^12^CO_2_ stretch (|1_3_⟩)	2364(3283)	2350(480)	2396(631)	2294(3198)	2340(3349)	2344.41
|2_2_1_3_⟩	3613–3638(0)	3639	3730	3507(21)	3598(21)	3599
|1_1_1_3_⟩	3681–3695(0)	3653	3743	3620(27)	3710(27)	3708
MAE^a^	–	–	–	58	4	

a Wavenumbers are reported in cm^–1^, while IR intensities (in parentheses) are in km/mol.

b Mean absolute error (in cm^–1^) of the theoretical data with respect to the experimental ones.

The transition moments were subsequently evaluated to compute the full IR spectrum, with the harmonic correction applied to fundamentals having a limited impact in this case. A full comparison between the harmonic and anharmonic IR spectra of dry ice and the experimental data is reported in Figure 1.

**Figure 1:**
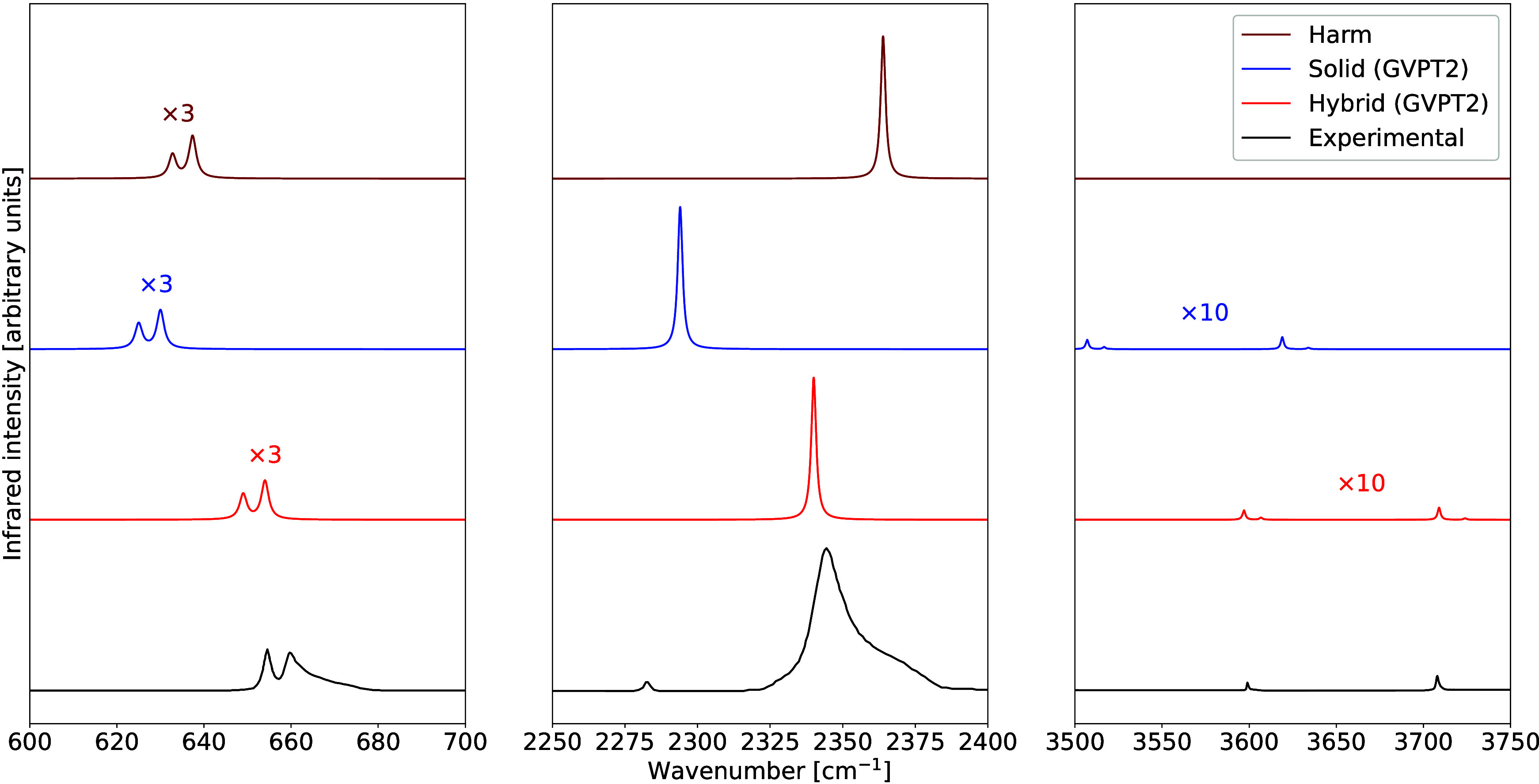
Comparison between theoretical and experimental IR spectra of dry ice at 15 K.

Although the hybrid GVPT2 approach yields excellent agreement with the experimental data in terms of vibrational band positions and splitting patterns, the accuracy of the computed IR intensities is not fully satisfactory. This discrepancy may have multiple origins. While dual-level methods benefit from high-level corrections to the harmonic frequencies, the transition dipole moments and hence the IR intensities are evaluated entirely within the solid-state framework at the PBE level, except for fundamental bands, whose corrections are negligible in this context. Given that infrared intensities are highly sensitive to the shape of the dipole moment surface as well as to anharmonic and resonant couplings, even small inaccuracies in first-order dipole derivatives—or an incomplete treatment of intensity-borrowing effects—may lead to notable deviations from experiment.^48^ In particular, the linear expansion of the dipole moment operator and the associated loss of information due to the neglect of higher-order terms (see Section S4 of the Supporting Information) may fail to fully capture subtle coupling-induced intensity redistributions, especially for combination bands or strongly interacting states.

Despite these limitations, the dual-level methodology devised by integrating gas-phase and solid-state simulations provides an accurate spectral profile and paves the way for the development of more refined approaches for the quantitative determination of vibrational spectra in solid systems.

We have presented a generalized and computationally efficient VPT2-based framework for simulating the anharmonic vibrational spectra of molecular solids. By applying this approach to solid carbon dioxide, we demonstrated its ability to accurately reproduce both fundamental transition energies and resonance-induced splittings, achieving close agreement with experimental IR data. The strategy adopted here, excluding resonant terms from the perturbative step and treating them more accurately in a subsequent variational step, ensures numerical stability while preserving essential vibrational couplings. This “perturb-then-diagonalize” formulation renders the method robust and broadly applicable, offering a practical alternative to more computationally demanding fully variational treatments.

The proposed computational protocol paves the way for reliable and scalable anharmonic simulations in the solid state with promising applications across a wide range of crystalline and molecular materials.

## Supplementary Material





## Notes

Flowchart of the computational approach; input deck for Crystal calculations; and equations of the VPT2 transition moments from the ground states up to three-quanta states (PDF)
Transparent Peer Review report available (PDF)
