# Supplementary material for: Anharmonicity in Molecular Crystals: Generalized Perturbation Theory Meets Periodic Computations
Source: J Phys Chem Lett. 2025 Sep 15;16(38):9956–62. doi: 10.1021/acs.jpclett.5c02217 (PMC12478864; doi:10.1021/acs.jpclett.5c02217)
Supplement: Supplementary file 1 [file jz5c02217_si_001.pdf]

Supporting Information
Anharmonicity in Molecular Crystals: Generalized Perturbation Theory Meets Periodic Computations

Davide Mitoli,[†] Alessandro Erba,[†] Vincenzo Barone,[‡] and Marco Mendolicchio[*,¶]

†*Università di Torino, Via Pietro Giuria 7, 10125, Torino (Italy)*

‡*INSTM, via G. Giusti 9, 50121 Firenze (Italy)*

¶*Scuola Normale Superiore, Piazza dei Cavalieri 7, 56126, Pisa (Italy)*

E-mail: marco.mendolicchio@sns.it

Contents

Section S1: Computational workflow

Section S2: Input deck for CRYSTAL calculations

Section S3: Full expressions of the transition moments (1, 2, and 3 quanta)

Section S4: Electrical Anharmonicity for the isolated molecule

S1 Computational workflow

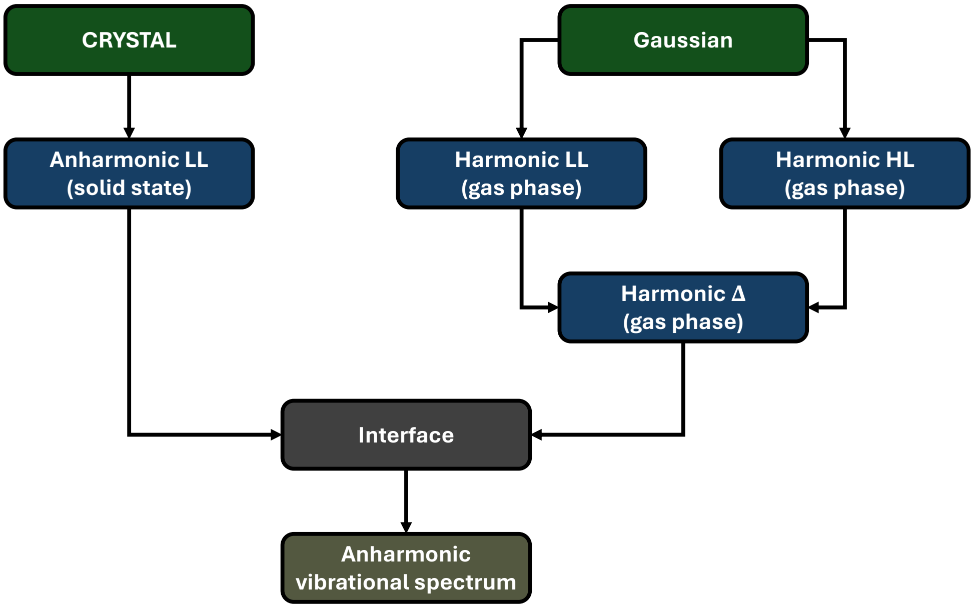

Figure 1: Computational engine for the calculation of solid-state vibrational spectra developed in this work.

S2 Input deck for Crystal calculations

```
CO2 solid PBE-D3/pob-TZVP-rev2 - PES
CRYSTAL
0 0 0
205
5.67747679
2
6      4.731503998356E-20  0.000000000000E+00  0.000000000000E+00
8      1.189359237113E-01  1.189359237113E-01  1.189359237113E-01
FREQCALC
NUMDERIV
2
ANHAPES
16
21 22 23 24 25 26 27 28 29 30 31 32 33 34 35 36
3 0.9
3MODTERM
END
END
6 10
0 0 6 2.0 1.0
  13575.3496820               0.22245814352D-03
   2035.2333680               0.17232738252D-02
    463.22562359              0.89255715314D-02
    131.20019598              0.35727984502D-01
     42.853015891             0.11076259931
     15.584185766             0.24295627626
0 0 2 2.0 1.0
      6.2067138508            0.41440263448
      2.5764896527            0.23744968655
0 0 1 0 1.0
      0.57696339419           1.0000000
0 0 1 0 1.0
      0.22972831358           1.0000000
0 2 4 2.0 1.0
     34.697232244             0.53333657805D-02
      7.9582622826            0.35864109092D-01
      2.3780826883            0.14215873329k10
END
DFT
PBE-D3
END
SHRINK
6 6
TOLINTEG
8 8 8 8 16
TOLDEE
10
END
```

S3 Transition moments

1-quanta transitions: fundamentals

$$\langle \boldsymbol{P} \rangle_{0,1_i} = s_0 \times S \times \boldsymbol{P}_i + \frac{s_2}{2}\sum_{j=1}^{N}\{\boldsymbol{P}_{jij} + \boldsymbol{P}_{ijj} + S\boldsymbol{P}_{jji}\} - \frac{s_0}{8}\sum_{j=1}^{N}\sum_{k=1}^{N}\mathsf{f}_{ijkk}\boldsymbol{P}_j\left[\frac{1}{\omega_i+\omega_j} - \frac{S(1-\delta_{ij})}{\omega_i-\omega_j}\right]$$
$$- \frac{s_1}{8}\sum_{j=1}^{N}\sum_{k=1}^{N}\left\{\mathsf{f}_{ijk}\left(\boldsymbol{P}_{jk}+\boldsymbol{P}_{kj}\right)\left(\frac{1}{\omega_i+\omega_j+\omega_k} - \frac{S}{\omega_i-\omega_j-\omega_k}\right) + \frac{\mathsf{f}_{jkk}}{\omega_j}\left[2S\boldsymbol{P}_{ji} + (1+S)\boldsymbol{P}_{ij}\right]\right\}$$
$$+ \frac{s_0}{2}\sum_{j=1}^{N}\sum_{k=1}^{N}\left(\sum_{\tau}B_{\tau}^{\mathrm{eq}}\zeta_{ik,\tau}\zeta_{jk,\tau}\right)\boldsymbol{P}_j\left\{\frac{\sqrt{\omega_i\omega_j}}{\omega_k}\left(\frac{1}{\omega_i+\omega_j} + \frac{S(1-\delta_{ij})}{\omega_i-\omega_j}\right) - \frac{\omega_k}{\sqrt{\omega_i\omega_j}}\left(\frac{1}{\omega_i+\omega_j} - \frac{S(1-\delta_{ij})}{\omega_i-\omega_j}\right)\right\}$$
$$+ \frac{s_0}{16}\sum_{j=1}^{N}\sum_{k=1}^{N}\sum_{l=1}^{N}\mathsf{f}_{ikl}\mathsf{f}_{jkl}\boldsymbol{P}_j\left\{(1-\delta_{ij})(1-\delta_{ik})(1-\delta_{il})\left[\frac{1}{(\omega_i+\omega_j)(\omega_i+\omega_k+\omega_l)}\right.\right.$$
$$\left. - \frac{1}{(\omega_i+\omega_j)(\omega_i-\omega_k-\omega_l)} - \frac{S}{(\omega_i-\omega_j)(\omega_i+\omega_k+\omega_l)} + \frac{S}{(\omega_i-\omega_j)(\omega_i-\omega_k-\omega_l)}\right]$$
$$+ \delta_{ij}(1+\delta_{ik})(1-\delta_{il})\left[\frac{1}{2\omega_i(\omega_i+\omega_k+\omega_l)} - \frac{1}{2\omega_i(\omega_i-\omega_k-\omega_l)} + \frac{S}{2(\omega_i+\omega_k+\omega_l)^2} - \frac{S}{2(\omega_i-\omega_k-\omega_l)^2}\right]$$
$$\left. + (1-\delta_{ij})(1-\delta_{ik})\delta_{il}\left[\frac{4}{\omega_k(\omega_i+\omega_j)} + \frac{2}{(\omega_i+\omega_j)(2\omega_i+\omega_k)} - \frac{4S}{\omega_k(\omega_i-\omega_j)} - \frac{2S}{(\omega_i-\omega_j)(2\omega_i+\omega_k)}\right]\right\}$$
$$+ \mathsf{f}_{ijk}\mathsf{f}_{llk}\boldsymbol{P}_j\left\{\frac{\delta_{ij}}{\omega_i\omega_k}\left(1 + \frac{\delta_{ik}\delta_{il}(6-4S)}{9}\right)\right.$$
$$+ (1-\delta_{ij})(1-\delta_{ik})(1-\delta_{il})\left[\frac{2}{\omega_k(\omega_i+\omega_j)} - \frac{2S}{\omega_k(\omega_i-\omega_j)}\right]$$
$$\left. + \delta_{ik}(1-\delta_{ij})(1+\frac{2\delta_{il}}{3})\left[\frac{2}{\omega_i(\omega_i+\omega_j)} - \frac{2S}{\omega_i(\omega_i-\omega_j)}\right]\right\}$$

2-quanta transitions: first overtones and combinations

$$\langle \boldsymbol{P} \rangle_{0,(1+\delta_{ij})_i(1-\delta_{ij})_j} = \sqrt{\frac{2}{1+\delta_{ij}}} \times \left[\frac{s_1 \times S}{2}(\boldsymbol{P}_{ij}+\boldsymbol{P}_{ji}) + \frac{s_0}{4}\sum_{k=1}^{N}\mathsf{f}_{ijk}\boldsymbol{P}_k\left(\frac{S}{\omega_i+\omega_j-\omega_k} - \frac{1}{\omega_i+\omega_j+\omega_k}\right)\right]$$

3-quanta transitions: second overtones and combinations

$$
\begin{aligned}
&\langle \boldsymbol{P} \rangle_{0,(1+\delta_{ij}+\delta_{ik})_i(1-\delta_{ij})_j(1-\delta_{ik})_k} = \\
&\quad s_2(\boldsymbol{P}_{ijk}+\boldsymbol{P}_{ikj}+\boldsymbol{P}_{jki})S \\
&\quad + A\sum_{l=1}^{N}\Bigg\{ \frac{s_0}{4}\mathsf{f}_{ijkl}\boldsymbol{P}_l \left[\frac{S}{\omega_i+\omega_j+\omega_k-\omega_l} - \frac{1}{\omega_i+\omega_j+\omega_k+\omega_l}\right] \\
&\qquad + \frac{s_0}{2}\sum_{\tau=x,y,z} B^{\mathrm{eq}}_{\tau}\boldsymbol{P}_l \Bigg\{ \Bigg[\zeta_{ij,\tau}\zeta_{kl,\tau}\left(\sqrt{\frac{\omega_j\omega_l}{\omega_i\omega_k}} - \sqrt{\frac{\omega_i\omega_l}{\omega_j\omega_k}}\right) + \zeta_{ik,\tau}\zeta_{jl,\tau}\left(\sqrt{\frac{\omega_k\omega_l}{\omega_i\omega_j}} - \sqrt{\frac{\omega_i\omega_l}{\omega_j\omega_k}}\right) \\
&\qquad\quad + \zeta_{il,\tau}\zeta_{jk,\tau}\left(\sqrt{\frac{\omega_k\omega_l}{\omega_i\omega_j}} - \sqrt{\frac{\omega_j\omega_l}{\omega_i\omega_k}}\right)\Bigg] \times \left[\frac{1}{\omega_i+\omega_j+\omega_k+\omega_l} + \frac{S}{\omega_i+\omega_j+\omega_k-\omega_l}\right] \\
&\qquad + \Bigg[\zeta_{ij,\tau}\zeta_{kl,\tau}\left(\sqrt{\frac{\omega_i\omega_k}{\omega_j\omega_l}} - \sqrt{\frac{\omega_j\omega_k}{\omega_i\omega_l}}\right) + \zeta_{ik,\tau}\zeta_{jl,\tau}\left(\sqrt{\frac{\omega_i\omega_j}{\omega_k\omega_l}} - \sqrt{\frac{\omega_j\omega_k}{\omega_i\omega_l}}\right) \\
&\qquad\quad + \zeta_{il,\tau}\zeta_{jk,\tau}\left(\sqrt{\frac{\omega_i\omega_j}{\omega_k\omega_l}} - \sqrt{\frac{\omega_i\omega_k}{\omega_j\omega_l}}\right)\Bigg] \times \left[\frac{1}{\omega_i+\omega_j+\omega_k+\omega_l} - \frac{S}{\omega_i+\omega_j+\omega_k-\omega_l}\right]\Bigg\} \\
&\qquad + \frac{s_1}{4}\Bigg\{ \mathsf{f}_{ijl}\left[\frac{S(\boldsymbol{P}_{kl}+\boldsymbol{P}_{lk})}{\omega_i+\omega_j-\omega_l} - \frac{\boldsymbol{P}_{kl}+S\boldsymbol{P}_{lk}}{\omega_i+\omega_j+\omega_l}\right] + \mathsf{f}_{ikl}\left[\frac{S(\boldsymbol{P}_{jl}+\boldsymbol{P}_{lj})}{\omega_i+\omega_k-\omega_l} - \frac{\boldsymbol{P}_{jl}+S\boldsymbol{P}_{lj}}{\omega_i+\omega_k+\omega_l}\right] \\
&\qquad\quad + \mathsf{f}_{jkl}\left[\frac{S(\boldsymbol{P}_{il}+\boldsymbol{P}_{li})}{\omega_j+\omega_k-\omega_l} - \frac{\boldsymbol{P}_{il}+S\boldsymbol{P}_{li}}{\omega_j+\omega_k+\omega_l}\right]\Bigg\} \\
&\qquad + \frac{s_0}{8}\sum_{m=1}^{N} \mathsf{f}_{ijm}\mathsf{f}_{klm}\boldsymbol{P}_l\Bigg[\frac{1}{(\omega_m+\omega_j+\omega_i)(\omega_l+\omega_k+\omega_j+\omega_i)} + \frac{1}{(\omega_m-\omega_j-\omega_i)(\omega_l+\omega_k+\omega_j+\omega_i)} \\
&\qquad\quad + \frac{S}{(\omega_m+\omega_j+\omega_i)(\omega_l-\omega_k-\omega_j-\omega_i)} + \frac{S}{(\omega_m-\omega_j-\omega_i)(\omega_l-\omega_k-\omega_j-\omega_i)}\Bigg] \\
&\qquad + \mathsf{f}_{ikm}\mathsf{f}_{jlm}\boldsymbol{P}_l\Bigg[\frac{1}{(\omega_m+\omega_k+\omega_i)(\omega_l+\omega_k+\omega_j+\omega_i)} + \frac{1}{(\omega_m-\omega_k-\omega_i)(\omega_l+\omega_k+\omega_j+\omega_i)} \\
&\qquad\quad + \frac{S}{(\omega_m+\omega_k+\omega_i)(\omega_l-\omega_k-\omega_j-\omega_i)} + \frac{S}{(\omega_m-\omega_k-\omega_i)(\omega_l-\omega_k-\omega_j-\omega_i)}\Bigg] \\
&\qquad + \mathsf{f}_{jkm}\mathsf{f}_{ilm}\boldsymbol{P}_l\Bigg[\frac{1}{(\omega_m+\omega_k+\omega_j)(\omega_l+\omega_k+\omega_j+\omega_i)} + \frac{1}{(\omega_m-\omega_k-\omega_j)(\omega_l+\omega_k+\omega_j+\omega_i)} \\
&\qquad\quad + \frac{S}{(\omega_m+\omega_k+\omega_j)(\omega_l-\omega_k-\omega_j-\omega_i)} + \frac{S}{(\omega_m-\omega_k-\omega_j)(\omega_l-\omega_k-\omega_j-\omega_i)}\Bigg]\Bigg\}
\end{aligned}
$$

with

$$A = \frac{\sqrt{1+\delta_{ij}+\delta_{ik}}}{(1+\delta_{ij}+\delta_{ik})!}$$

and

$$\langle \boldsymbol{P} \rangle_{(1+\delta_{ij}+\delta_{ik})_i(1-\delta_{ij})_j(1-\delta_{ik})_k,0} = S \times \langle \boldsymbol{P} \rangle_{0,(1+\delta_{ij}+\delta_{ik})_i(1-\delta_{ij})_j(1-\delta_{ik})_k}$$

S4 Anharmonic analysis of isolated carbon dioxide (gas phase)

Table 1: Anharmonic transition frequencies (in cm^{-1}) and intensities (in Km/mol) of carbon dioxide at the rDSD level of theory. Only transitions whose intensity exceeds 1 Km/mol are reported.

ν[a]	I (Red)[b]	I (Full)[c]
662	27	26
662	27	26
2352	622	605
3597	3	6
3705	6	11

[a] GVPT2 anharmonic wavenumber. [b] Intensity obtained by neglecting the second- and third-order derivatives of the dipole moment with respect to the normal coordinates. [c] Intensity obtained by including up to the semi-diagonal third-order derivatives of the dipole moment.
