# Supplementary material for: Anharmonicity in Molecular Crystals: Generalized Perturbation Theory Meets Periodic Computations
Source: J Phys Chem Lett. 2025 Sep 15;16(38):9956–62. doi: 10.1021/acs.jpclett.5c02217 (PMC12478864; doi:10.1021/acs.jpclett.5c02217)
Supplement: Supplementary file 2 [file jz5c02217_si_002.pdf]

jz-2025-02217g.R1

Name: Peer Review Information for "Anharmonicity in Molecular Crystals: Generalized Perturbation Theory Meets Periodic Computations"

First Round of Reviewer Comments

Reviewer: 1

Comments to the Author

The work authored by D. Mitoli et al. consists in an original theoretical contribution to describe the vibrational spectra of molecules at the solid state. The topic is of general interest, since many systems, at normal conditions, exist as solid crystal or molecular solids whose vibrational spectrum needs a computational help in order to be interpreted and fully rationalized. This problem is still challenging for theory, since many different issues must be considered in order to obtain a robust protocol: 1) anharmonicity 2) intermolecular interactions 3) resonance terms. Moreover, a subtle balanced scheme must be employed to obtain harmonic and anharmonic terms. Harmonic terms are leading ones and need high level electronic structure scheme to obtain quantitative agreement, on the other hand anharmonic terms are smaller and lower level schemes can be safely employed to obtain sounding results. The present work solves the problem in a very elegant and efficient way: the intermolecular interactions are included due to the periodic calculations employed (with CRYSTAL code), the anharmonicity terms are explicitly included in the model, and finally, the resonant terms which blow up some perturbative terms due to zero denominators are avoided due to their suppression in the perturbative treatment and variationally considered by a successive re-diagonalization of the Hamiltonian. The new theoretical scheme is very well explained and implemented, and also the electronic structure choice (level of DFT methods as well as basis set size) are justified. The final results for dry ice (solid CO2) are critically compared with the experiment giving excellent agreement, allowing a robust assignment of bands and also a clear understanding of the specific role of different physical effects (anharmonicity, interactioncs, etc.).

In summary the paper is very interesting for a wide scientific audience, is very well written and the conclusions are convincing. Therefore I recommend publication in its present form in JPCL.

Reviewer: 2

Comments to the Author

Manuscript introduces computational framework for anharmonic vibrational spectra simulations in the solid state combining capacities of widely used computational packages CRYSTAL and GAUSSIAN. This combination allows to take advantage of CRYSTAL ability to compute anharmonic force field for periodic systems at DFT level, with GVPT2 computations and double hybrid DFT (DH-DFT) harmonic computations available in Gaussian.

That is significant, important and I believe well welcome development within computational spectroscopy, which will improve analysis and understanding of IR spectra recorded for solid state samples.

Proposed methodology is applied to the CO2 crystal, small molecule which is well known for strong anharmonic effects due to Fermi resonance, showing very good agreement with experiment also for non-fundamental bands.

Considering that GVPT2 computations especially in conjunction with hybrid DH-DFT/DFT proved to be very effective in simulation IR spectra for isolated molecules, it can be expected that extension of this methodology to periodic, solid state system will represent important advance allowing to simulate directly spectra of similar systems in the solid state, without the need of introducing approximate yet elaborate approaches as for instance cluster models (i.e. DOI: 10.1021/jp510101y).

The manuscript should be improved by clarifying better the details on the whole workflow. So far SI reports sample input file for CRYSTAL. Next it is stated that "the resulting quantities have then been processed by a standalone module" - is the module available somewhere? Is this module a link to Gaussian? All references about VPT2 and GVPT2 refers

to the implementation within Gaussian, so mentioning "standalone module" is confusing. In particular it is not clear if similar computations can be performed using Gaussian as GVPT2 engine (with some local modifications?) or require other code.

Minor comments

1. Notation used for assignment should be described in Table 1 or elsewhere.

2. For the discussion on IR intensities starting in lines 44 on page 11 sentence "Given that infrared intensities are highly sensitive to the shape of the dipole moment surface as well as to anharmonic and resonant couplings, even small inaccuracies in first-order dipole derivatives—or an incomplete treatment of intensity borrowing effects—may lead to notable deviations from experiment." can be supported by recent reference dealing with this topic (DOI: 10.1021/acs.jpca.5c02226).

Author's Response to Peer Review Comments:

Reviewer: 1

Recommendation: This paper represents a significant new contribution and should be published as is.

Comments:

The work authored by D. Mitoli et al. consists in an original theoretical contribution to describe the vibrational spectra of molecules at the solid state. The topic is of general interest, since many systems, at normal conditions, exist as solid crystal or molecular solids whose vibrational spectrum needs a computational help in order to be interpreted and fully rationalized. This problem is still challenging for theory, since many different issues must be considered in order to obtain a robust protocol: 1) anharmonicity 2) intermolecular interactions 3) resonance terms. Moreover, a subtle balanced scheme must be employed to obtain harmonic and anharmonic terms. Harmonic terms are leading ones and need high level electronic structure scheme to obtain quantitative agreement, on the other hand anharmonic terms are smaller and lower level schemes can be safely employed to obtain sounding results. The present work solves the problem in a very elegant and efficient way: the intermolecular interactions are included due to the periodic calculations employed (with CRYSTAL code), the anharmonicity terms are explicitly included in the

model, and finally, the resonant terms which blow up some perturbative terms due to zero denominators are avoided due to their suppression in the perturbative treatment and variationally considered by a successive re-diagonalization of the Hamiltonian. The new theoretical scheme is very well explained and implemented, and also the electronic structure choice (level of DFT methods as well as basis set size) are justified. The final results for dry ice (solid CO2) are critically compared with the experiment giving excellent agreement, allowing a robust assignment of bands and also a clear understanding of the specific role of different physical effects (anharmonicity, interactioncs, etc.).

In summary the paper is very interesting for a wide scientific audience, is very well written and the conclusions are convincing. Therefore I recommend publication in its present form in JPCL.

Additional Questions:

Urgency: Top 10%

Significance: Top 10%

Novelty: Top 10%

Scholarly Presentation: Top 10%

Is the paper likely to interest a substantial number of physical chemists, not just specialists working in the authors' area of research?: Yes

Reviewer: 2

Recommendation: This paper is publishable subject to minor revisions noted.
Further review is not needed.

Comments:

Manuscript introduces computational framework for anharmonic vibrational spectra simulations in the solid state combining capacities of widely used computational packages CRYSTAL and GAUSSIAN. This combination allows to take advantage of CRYSTAL ability to compute anharmonic force field for periodic systems at DFT level, with GVPT2 computations and double hybrid DFT (DH-DFT) harmonic computations available in Gaussian.

That is significant, important and I believe well welcome development within computational spectroscopy, which will improve analysis and understanding of IR spectra recorded for solid state samples.

Proposed methodology is applied to the CO2 crystal, small molecule which is well known for strong anharmonic effects due to Fermi resonance, showing very good agreement with experiment also for non-fundamental bands.

Considering that GVPT2 computations especially in conjunction with hybrid DHDFT/DFT proved to be very effective in simulation IR spectra for isolated molecules, it can be expected that extension of this methodology to periodic, solid state system will represent important advance allowing to simulate directly spectra of similar systems in the solid state, without the need of introducing approximate yet elaborate approaches as for instance cluster models (i.e. DOI: 10.1021/jp510101y).

The manuscript should be improved by clarifying better the details on the whole workflow.

So far SI reports sample input file for CRYSTAL. Next it is stated that "the resulting quantities have then been processed by a standalone module" - is the module available somewhere? Is this module a link to Gaussian? All references about VPT2 and GVPT2 refers to the implementation within Gaussian, so mentioning

"standalone module" is confusing. In particular it is not clear if similar computations can be performed using Gaussian as GVPT2 engine (with some local modifications?) or require other code.

Further details about the overall workflow have been included. In general terms, the external program features an interface that can extract data from a standard CRYSTAL output file and directly apply VPT2 to obtain the anharmonic spectrum. An additional interface enables the extraction of harmonic frequencies from a GAUSSIAN 16 output file to apply the hybrid model.

Minor comments

1. Notation used for assignment should be described in Table 1 or elsewhere. The notation used for assignment has been explicitly described.

2. For the discussion on IR intensities starting in lines 44 on page 11 sentence "Given that infrared intensities are highly sensitive to the shape of the dipole moment surface as well as to anharmonic and resonant couplings, even small inaccuracies in first-order dipole derivatives—or an incomplete treatment of intensity borrowing effects—may lead to notable deviations from experiment." can be supported by recent reference dealing with this topic (DOI:

10.1021/acs.jpca.5c02226).

The suggested reference has been included.

Additional Questions:

Urgency: High

Significance: High

Novelty: Top 10%

Scholarly Presentation: High

Is the paper likely to interest a substantial number of physical chemists, not just specialists working in the authors' area of research?: Yes
